# Left thoracotomy for coronary artery bypass grafting after sternoturnover for pectus excavatum: a case report

**DOI:** 10.1186/s40792-019-0692-8

**Published:** 2019-08-16

**Authors:** Mamoru Hamuro, Kenji Yamamoto, Tomoyuki Yamada, Sakae Enomoto

**Affiliations:** Department of Cardiovascular Surgery, Okamura Memorial Hospital, 293-1 Kakita, Shimizu, Sunto District, Shizuoka Prefecture 411-0904 Japan

**Keywords:** Sternoturnover, Pectus excavatum, Thoracotomy, Coronary artery bypass grafting

## Abstract

**Background:**

Sternoturnover is a surgical procedure for pectus excavatum. Cardiac surgery in patients with a history of sternoturnover has been rarely reported and is a surgical challenge because it is unknown how median sternotomy or the use of a sternal retractor affects the postoperative stability of the thorax and respiratory function. We report a successful coronary artery bypass grafting through left thoracotomy in a patient treated with sternoturnover for pectus excavatum.

**Case presentation:**

A 53-year-old man, who underwent sternoturnover in his childhood, was diagnosed with acute myocardial infarction, and percutaneous coronary intervention was performed as the acute treatment of the culprit lesion. Because residual lesions were present, he was referred to our department for coronary artery bypass grafting. Enhanced computed tomography revealed bilateral occlusions of the internal thoracic arteries and a small fragile sternum after fixation. Considering postoperative respiratory dysfunction associated with instability of the thorax following median sternotomy, we selected left thoracotomy for coronary artery bypass grafting. Convalescence was uneventful without any respiratory complications.

**Conclusion:**

Left thoracotomy is useful for coronary artery bypass grafting in patients previously treated with sternoturnover for pectus excavatum because it can avoid respiratory dysfunction associated with median sternotomy.

## Background

Sternoturnover is a surgical procedure for pectus excavatum. Cardiac surgery in patients with a history of sternoturnover has been rarely reported and is a surgical challenge because it is unknown how median sternotomy or the use of a sternal retractor affects the postoperative stability of the thorax and respiratory function. We report a successful coronary artery bypass grafting (CABG) through left thoracotomy in a patient treated with sternoturnover for pectus excavatum.

## Case presentation

**Fig. 1:**
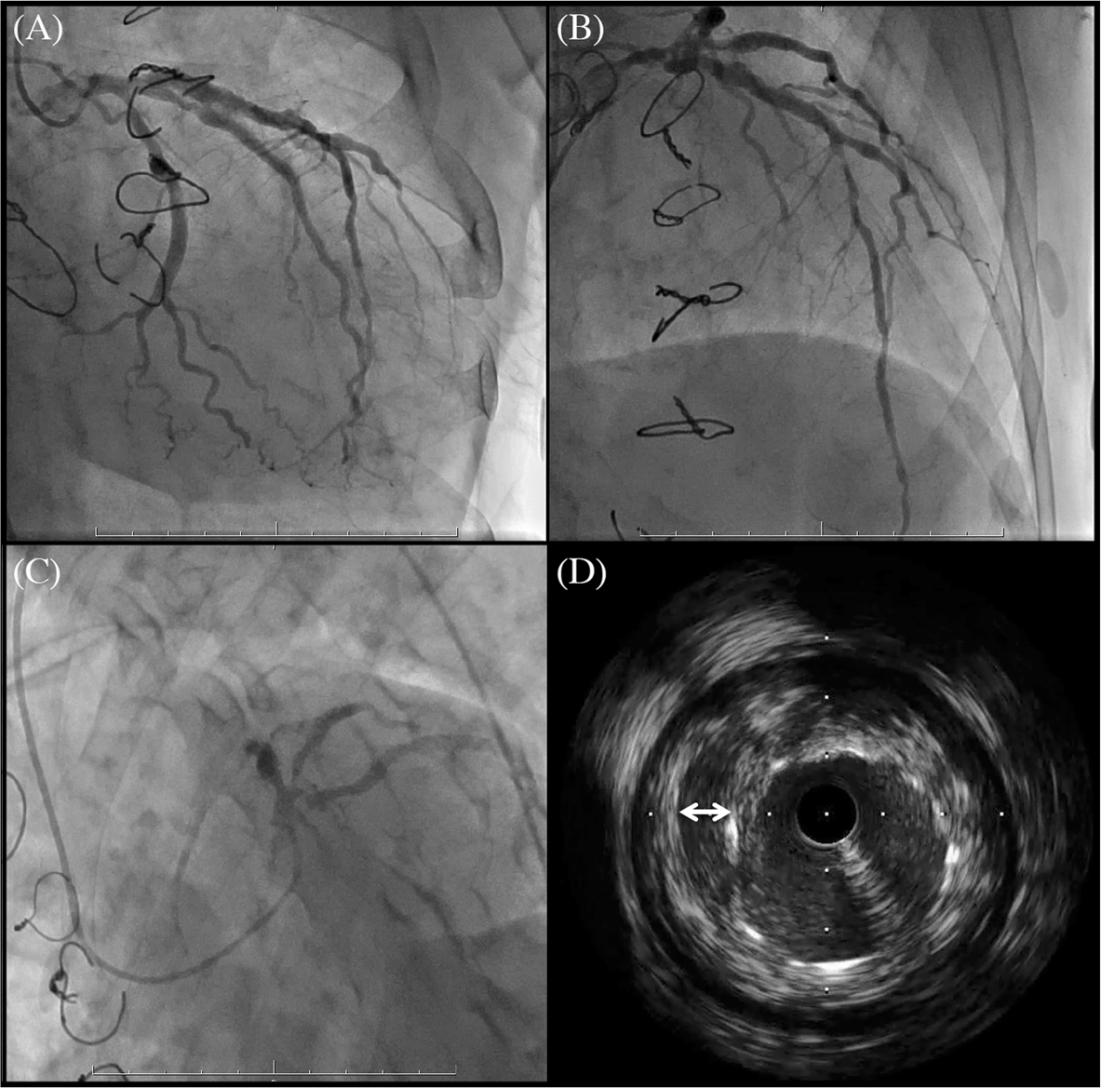
Preoperative coronary angiography (**a**–**c**) shows stenoses of the left anterior descending artery, high lateral branch, and left circumflex artery. Intravascular ultrasound (**d**) shows a stent malapposition at the site of the coronary aneurysm

**Fig. 2:**
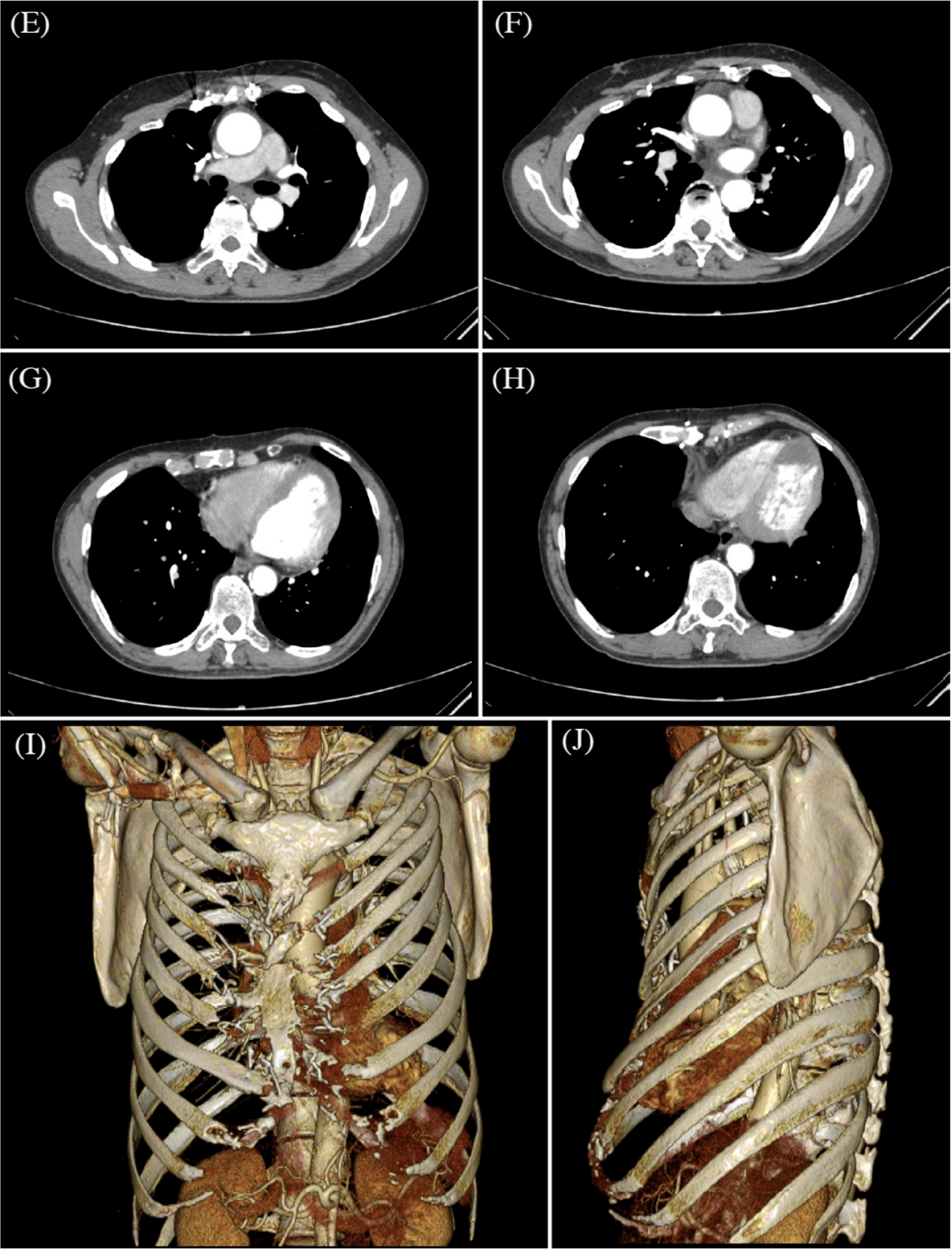
Preoperative axial (**e**–**h**) and three-dimensional (**i**, **j**) computed tomography shows bilateral occlusion of the internal thoracic arteries and a small fragile sternum after sternoturnover fixation

A 53-year-old man, who had undergone sternoturnover for pectus excavatum as a child, was diagnosed with acute myocardial infarction, and percutaneous coronary intervention was performed as the acute treatment of the culprit lesion. Two drug-eluting stents were implanted at the aneurysmal site of the left anterior descending artery and the high lateral branch. However, coronary angiography after coronary intervention demonstrated residual stenoses of the left anterior descending artery, high lateral branch, and left circumflex artery, and intravascular ultrasound revealed stent malappositions at the sites of the coronary aneurysm (Fig. 1). Because the duration of patency of the stent implanted within the coronary artery aneurysm was unpredictable and a residual lesion was present, he was referred to our department for CABG. Echocardiogram showed left ventricular dysfunction with an ejection fraction of 19%. Enhanced computed tomography revealed bilateral occlusions of the internal thoracic arteries and a small fragile sternum after sternoturnover fixation (Fig. 2). Considering postoperative respiratory dysfunction associated with instability of the thorax following median sternotomy, low left ventricular function, and left coronary artery as the only target vessel, we selected left thoracotomy for CABG without cardiopulmonary bypass.

**Fig. 3:**
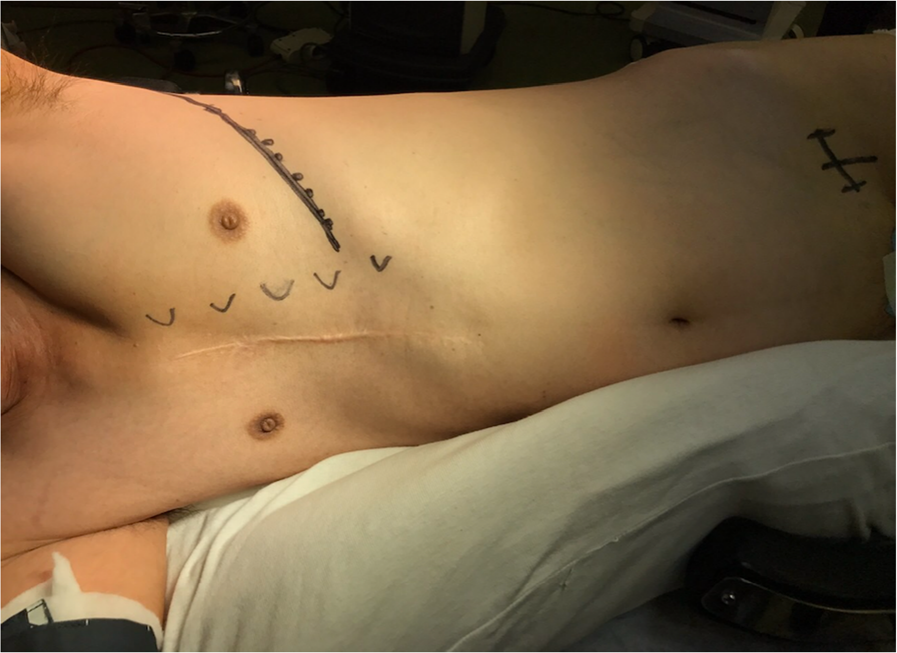
Operative position view of semi right lateral decubitus position. The picture shows deformation of the thorax around the sternum

**Fig. 4:**
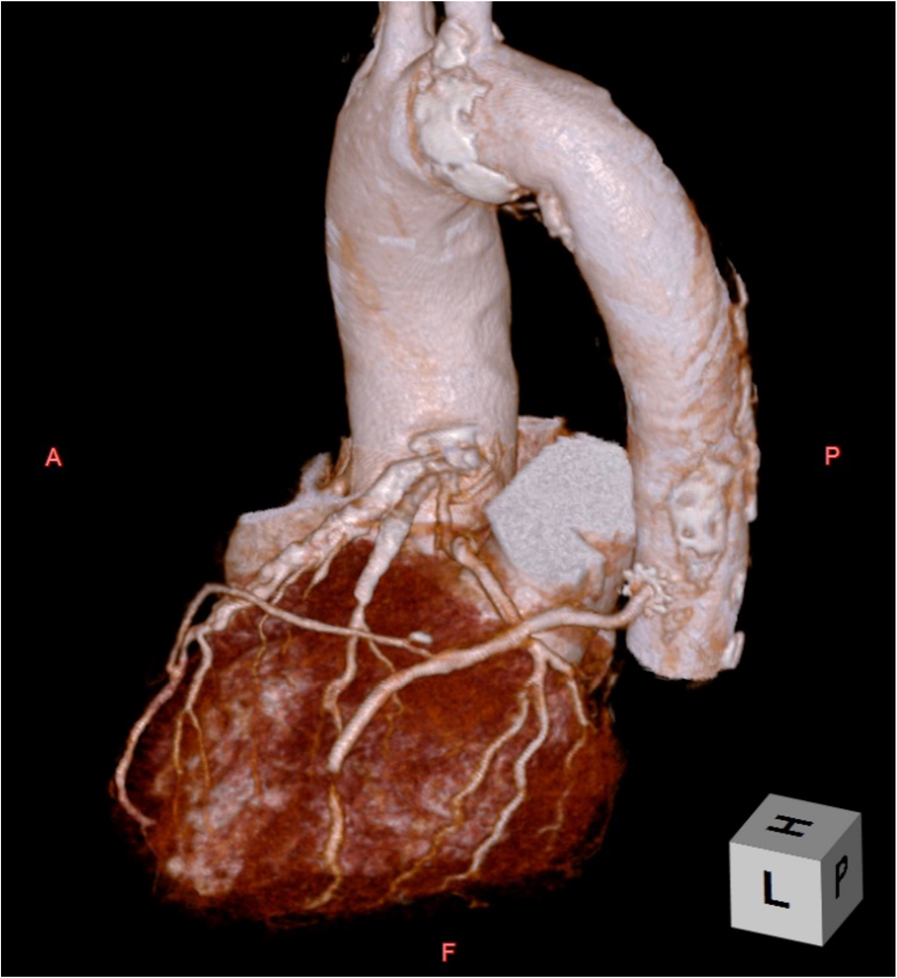
Postoperative three-dimensional coronary computed tomography angiography shows patent bypass grafts

The left radial artery and great saphenous vein were harvested with the patient lying supine under general anesthesia. He was then placed in a semi right lateral decubitus position (Fig. 3). The thoracotomy was made by a left anterolateral incision at the fifth intercostal space. The pulmonary ligament was dissected to open a route for the grafts. The pericardium was opened posterior to the left phrenic nerve, and the target vessels were exposed. After systemic heparinization, the saphenous vein graft was anastomosed to the descending aorta using the PAS-Port System (Cardica Inc., Redwood City, CA, USA). The venous graft was sequentially anastomosed to the left circumflex artery and high lateral branch. The radial artery graft was anastomosed proximally to the venous graft and distally to the left anterior descending artery. The patient had an uneventful recovery and was discharged on postoperative day 12. Postoperative enhanced computed tomography proved all bypass grafts patent (Fig. 4).

## Discussion

Pectus excavatum is a congenital chest deformity affecting the costal cartilages, and in some cases, it is surgically treated with sternoturnover. Cardiac surgery after sternoturnover is challenging because it is unclear how median sternotomy and sternal retractor affects postoperative thorax stability and respiratory function in patients with a history of sternoturnover. Although there are some reports of the first sternotomy for cardiac surgery in a patient with pectus excavatum [1] and simultaneous cardiac surgery and repair of pectus excavatum [2], median sternotomy after sternoturnover has been rarely reported. Mohan et al. [3] performed CABG through sternotomy after sternal elevation with metal implant and noted that some surgeons recommend avoiding sternotomy as a repeat approach to the mediastinum. Kanaoka et al. [4] reported a case of median sternotomy for aortic surgery 13 years after sternoturnover, followed by flail chest and 15-day mechanical ventilation. Sasaki et al. [5] reported a case of aortic surgery with median sternotomy after sternoturnover, in which the sternum was retracted like a trap door instead of using a sternal retractor. Despite this precaution, the patient needed a tracheostomy and 23-day postoperative ventilator support. Our patient experienced sternoturnover in childhood. The small, reversed sternum and the costal cartilages appeared premature and fragile, leading to concerns of instability of the thorax and respiratory dysfunction after median sternotomy. Left thoracotomy was thus preferred for this patient.

Left thoracotomy is recommended for CABG in patients with sternoturnover because it reduces the risk of adverse effects on the thorax. Left thoracotomy has been used for repeat CABG procedures because it reduces the risk of injury to the heart or previous patent grafts during dissection of adhesions [6]. There are previous reports of left thoracotomy for cardiac surgery in patients with pectus excavatum. Bastidas et al. [7] reported left thoracotomy for mitral valve replacement in a patient with pectus excavatum and recommended that approach, particularly for severe pectus excavatum with the heart displaced to the left. Choghari et al. [8] reported left thoracotomy for CABG in a patient with pectus excavatum, noting that it offers excellent exposure of the anterolateral aspect of the heart and access of the internal thoracic artery. Although these reports did not involve patients after sternoturnover, left thoracotomy may provide favorable surgical field in some cases with a history of sternoturnover because the heart may still deviates to the left. Conversely, because the length between the sternum and the spine may be shorter in such patients and pulmonary artery locates in front of the proximal ascending aorta via left thoracotomy, it may be difficult to ensure surgical field of the proximal ascending aorta and need consideration for bypass graft design. Nevertheless, left thoracotomy is considered to be an optimal approach in point of avoiding adverse effects on the thorax caused by median sternotomy. In our patient, the left coronary artery was the only target vessel; therefore, we chose off-pump CABG. In patients with right coronary artery or three-vessel disease, it may be necessary to consider other options, such as transdiaphragmatic approach, or using cardiopulmonary bypass.

## Conclusion

In patients previously treated with sternoturnover, left thoracotomy is useful for CABG to avoid risks of thorax instability and respiratory complications after median sternotomy.

## Data Availability

The dataset supporting the conclusions of this article is included within the article.

## Abbreviation

CABG: Coronary artery bypass grafting

## References

[1] Yang Z, Yang S, Wang F, Wang C (2016). Acute aortic dissection in pregnant women. Gen Thorac Cardiovasc Surg.

[2] Tuncer E, Vuran AC, Ozyuksel A, Yeginsu A, Ceyran H (2017). Simultaneous repair of pectus excavatum and pulmonary valve implantation years after complete repair of tetralogy Fallot. Gen Thorac Cardiovasc Surg.

[3] Mohan PS, Stark RD, Costic JT, Seinfeld FI, Laub GW (2005). Coronary artery bypass via resternotomy after pectus excavatum repair. Am Surg.

[4] Kanaoka Y, Tanemoto K, Murakami T, Kuroki K, Minami H, Kuinose M (2001). Operation for acute aortic dissection 13 wears after operation for funnel chest in Marfan syndrome. Jpn J Cardiovasc Surg.

[5] Sasaki H, Aomi S, Noji S (2001). A re-operative case of bentall operation and aortic arch replacement using a stent graft for Marfan syndrome, post sternum turnover and post mitral valve replacement. Kyobu Geka.

[6] Takahashi K, Takeuchi S, Ito K, Chiyoya M, Kondo N, Minakawa M (2012). Reoperative coronary artery bypass surgery: avoiding repeat median sternotomy. Ann Thorac Surg.

[7] Bastidas JG, Razzouk AJ, Hasaniya NW, Bailey LL (2013). Left thoracotomy: an ideal approach for mitral valve replacement in patient with severe chest wall deformity. Ann Thorac Surg.

[8] Choghari C, Heymans O, Geens M, Joris M (1996). Left thoracotomy for coronary bypass in a patient with pectus excavatum. Ann Thorac Surg.

